# Species identification and antimicrobial susceptibility testing of non-tuberculous mycobacteria isolated in Chongqing, Southwest China

**DOI:** 10.1017/S0950268820003088

**Published:** 2020-12-28

**Authors:** Huizheng Zhang, Ming Luo, Ke Zhang, Xin Yang, Kui Hu, Zongqiang Fu, Liwen Zhang, Ping Wu, Dongyong Wan, Mei Han, Xiaoying Wang

**Affiliations:** 1 Central Laboratory, Chongqing Public Health Medical Centre, Chongqing 400036, China; 2 School of Nursing, Southwest Medical University, Sichuan 646000, China; 3 Operation Room, Chongqing Emergency Medical Centre, Chongqing 400014 China; 4 Department of Cardiothoracic Surgery, The First Affiliated Hospital of Chongqing Medical University, Chongqing 400042, China; 5 Department of Paediatrics, Dazhou Central Hospital, Sichuan 635000, China; 6 Clinical Laboratory, Henan Province Hospital of Traditional Chinese Medicine, Henan 450002, China; 7 Department of Tuberculosis, Chongqing Public Health Medical Centre, Chongqing 400036, China; 8 Clinical Laboratory, Chongqing Public Health Medical Centre, Chongqing 400036, China; 9 Faculty of Medical Technology, Chongqing Medical and Pharmaceutical College, Chongqing 401331, China

**Keywords:** Antimicrobial susceptibility testing, Chongqing, *Mycobacterium abscessus complex*, non-tuberculous mycobacteria (NTM), species identification

## Abstract

With the rapid rise in the prevalence of non-tuberculous mycobacteria (NTM) diseases across the world, the microbiological diagnosis of NTM isolates is becoming increasingly important for the diagnosis and treatment of NTM disease. In this study, the clinical presentation, species distribution and drug susceptibility of patients with NTM disease visiting the Chongqing Public Health Medical Centre during March 2016–April 2019 were retrospectively analysed. Among the 146 patients with NTM disease, eight NTM species (complex) were identified. The predominant NTM species in these patients were identified to be *Mycobacterium abscessus complex* (53, 36.3%), *M. intracellulare* (38, 26%) and *M. fortuitum* (17, 11.7%). In addition, two or more species were isolated from 7.5% of the patients. Pulmonary NTM disease (142, 97.3%) showed the highest prevalence among the patients. It was observed that 40.1% of the patients with pulmonary NTM disease had chronic pulmonary obstructive disease and bronchiectasis, while 22.5% had prior tuberculosis. Male patients showed more association with the conditions of cough and haemoptysis than the female patients. In an *in vitro* antimicrobial susceptibility testing, most of the species showed susceptibility to linezolid, amikacin and clarithromycin, while *M. fortuitum* exhibited low susceptibility to tobramycin. In conclusion, the prevalence of NTM disease, especially that of the pulmonary NTM disease, is common in Southwest China. Species identification and drug susceptibility testing are thus extremely important to ensure appropriate treatment regimens for patient care and management.

## Background

Non-tuberculous mycobacteria (NTM) are environmental microbes that occur predominantly in water and soil. More than 160 NTM species have been identified until date [1], with at least 42 being implicated in infectious cases [2]. The incidence of NTM infections is increasing worldwide [3, 4]. In China alone, the incidence of NTM disease was found to increase from 4.9% in 1990 to 22.9% in 2010 among TB suspects [5]. The pathogens can infect a wide variety of tissues, and the majority (77.4–91.5%) of them are isolated from pulmonary specimens [6, 7]. The well-defined risk factors for this disease are the female gender, advanced age, immunodeficient status and chronic respiratory diseases [8–11]. Specifically, conditions such as bronchiectasis, chronic obstructive pulmonary disease (COPD) and tuberculosis (TB) are likely to predispose individuals to pulmonary NTM diseases [11]. However, its epidemiology has been difficult to establish as reporting of NTM infections is not mandatory in most regions of the world. Therefore, research and accumulation of precise epidemiological and surveillance data are urgently needed for the effective treatment of NTM patients.

The clinical manifestations of different mycobacteria infection may be similar, but the treatment regimens differ widely. In this context, accurate identification of the species is extremely important as the drug resistance profile of NTM is highly species-specific [12]. Macrolide-containing multidrug regimen has been regarded as the preferred treatment of choice for handling NTM infection [13]. However, drug susceptibility tests for NTM species have not been extensively conducted in China. Therefore, accurate information on antimicrobial susceptibility is essential for the clinicians to select appropriate therapeutic regimens.

Chongqing, with a population of about 32 million, is the only municipal city in Southwest China that has a high incidence of TB. However, clinical characteristics, species distribution and drug susceptibility of NTM isolates, which are extremely important for the diagnosis and treatment of NTM diseases, have not been conducted in this region. Thus, we retrospectively analysed the prevalence of NTM species and their drug resistance characteristics, which are envisaged to be useful in providing reference evidence for the control of NTM disease epidemic in this region.

## Methods

### Study design and population

This study was conducted at the Chongqing Public Health Medical Centre, a specialist hospital for TB and other infectious diseases in Chongqing, China. We retrospectively reviewed the medical records of patients with NTM diseases who were registered in the hospital during March 2016–April 2019. The demographic and clinical characteristics as well as the laboratory examination outcomes, including those for acid-fast bacillus (AFB) smear, molecular diagnostic testing and drug susceptibility testing, were assessed in this study. For patients with multiple isolates of one or more species, only the first isolate of the same species was included for further analysis. The NTM disease was defined as specified by the 2007 American Thoracic Society and the Infectious Diseases Society of America (AST/IDSA) [13] and further categorised as pulmonary NTM disease or extrapulmonary NTM disease. This study was approved by the Institutional Review Board of Chongqing Public Health Medical Centre. The ethics committee waived the requirement for written informed consent as all patient information used in this study had been routinely collected and were analysed anonymously.

### Bacterial culturing and identification

The clinical specimens, including bronchoalveolar lavage, pleural fluid, sputum specimens and extrapulmonary tissue, from the patients under the study for NTM disease were collected for preparing the AFB smear. Moreover, the samples were cultured on Lowenstein–Jensen (L-J) medium after treatment with 4% NaOH. Culture-positive isolates were tested for the expression of MPT64 protein, which is the main protein secreted by the *Mycobacterium tuberculosis* complex. The suspicious NTM colonies, which were MPT64-negative, were further identified by using the polymerase chain reaction (PCR)-reverse dot-blot hybridisation kit (Yaneng Bio, Shenzhen, China) as per the manufacturer's instruction. The PCR-reverse dot-blot hybridisation kit was designed to identify 21 NTM species and the *M. tuberculosis* complex. The 16S rDNA sequences and oligonucleotide probes that specifically distinguish different *Mycobacterium* sequences were used to identify the *Mycobacterium* up to the species level. Briefly, genomic DNA was prepared by adding 50 μl of lysis buffer, followed by direct boiling. The DNA was amplified in a specific tube containing the PCR mixture. Heat-denatured single-stranded PCR products were used to hybridise with the oligonucleotide probes on the membranes at 58 °C for 90 min. After adding tetramethylbenzidine and hydrogen peroxide, the results were read based on the appearance of visible purple-blue spots on the membrane. Finally, the results were interpreted as instructed by the manufacturer.

### Drug susceptibility testing

The *in vitro* antimicrobial susceptibility testing of NTM was performed by the broth microdilution method according to the Clinical and Laboratory Standards Institute (CLSI) guidelines [14]. Nine antimicrobial agents were enrolled in this study, including clarithromycin, amikacin, moxifloxacin, linezolid, cefoxitin, tobramycin, rifampicin, minocycline and rifabutin. The minimum inhibitory concentration (MIC) was defined as the lowest concentration of the drug that inhibited the visible growth of the tested isolates. The MIC breakpoints, namely sensitive, intermediate and resistant, were interpreted as suggested by the CLSI guidelines [14].

### Data collection

Patient information was acquired from the electronic patient medical records system of the Chongqing Public Health Medical Centre. The following data were collected: (1) demographic details, (2) clinical presentation, (3) radiological findings, (4) medical comorbidities and (5) microbiology results and other information, including any pre-existing lung diseases.

### Statistical analysis

SPSS software (IBM SPSS Statistics for Windows, Version 17.0; SPSS Inc., Chicago, IL, USA) was used for statistical analysis. Pearson *χ*^2^ test was employed for comparing the categorical variables. Statistically significant differences were considered when the *P* value was <0.05.

## Results

### Specimen source and NTM species identification

A total of 146 NTM patients were enrolled during March 2016–April 2019. Among the 146 patients with NTM disease, 142 (97.3%) harboured the isolates in their respiratory tract. Only in four patients (2.2%, 4/146), the isolates were from the extrapulmonary tissues: one from the cerebrospinal fluid; two from the skin/soft tissue; and one from urine sample. Eight species (complex) were identified in the patients, and the predominant ones were *M. abscessus complex* (53, 36.3%), *M. intracellulare* (38, 26%) and *M. fortuitum* (17, 11.7%). In 135 cases (92.5%), only a single species was isolated; however, in 11 cases (7.5%), ≥2 species were detected. The isolated species are listed in Table 1.

**Table 1.** NTM species distribution among 146 patients with NTM disease

Organism	No. of counts (%)
1 NTM species detected	135 (92.5)
*M. abscessus complex*	53 (36.3)
*M. intracellulare*	38 (26.0)
*M. fortuitum*	17 (11.7)
*M. gordonae*	11 (7.5)
*M. avium*	8 (5.5)
*M. kansasii*	6 (4.1)
*M. chelonae*	1 (0.7)
*M. xenopi*	1 (0.7)
≥2 NTM species detected	11 (7.5)
*M. intracellulare/avium complex*	3 (2.0)
*M. gordonae/fortuitum*	3 (2.0)
*M. intracellulare/abscessus complex*	2 (1.4)
*M. fortuitum/avium*	1 (0.7)
*M. abscessus complex/avium*	1 (0.7)
*M. abscessus complex/fortuitum*	1 (0.7)

### Demographic and clinical features of pulmonary NTM diseases

The demographic and clinical characteristics of pulmonary NTM patients are summarised in Table 2. The average age of the patients with pulmonary NTM disease was 46.8 years, and 88 (62.0%) of the patients were male. Furthermore, 125 patients (88.0%) exhibited cough, and 47 (33.1%) reported bronchiectasis as the major complication. Only 23.9% of the AFB smears were positive. The clinical features of male and female patients with pulmonary NTM disease were compared (Table 3). Male patients showed a significantly higher prevalence of cough and haemoptysis than the female patients.

**Table 2.** Baseline characteristics of patients with pulmonary NTM disease (*N* = 142)

Characteristics	No. of subjects (%)
Gender	
Male	88 (62.0)
Female	54 (38.0)
Average age (years)	46.8
Comorbidities	
TB history	32 (22.5)
COPD	10 (7.0)
Bronchiectasis	47 (33.1)
Malignant diseases	9 (6.3)
Diabetes mellitus	8 (5.6)
HIV	9 (6.3)
Clinical presentations	
Cough	125 (88.0)
Haemoptysis	44 (31.0)
Laboratory investigations	
CD4 (<200 cells/μl)	19 (13.4)
AFB smear (+)	34 (23.9)
Chest X-ray	
Fibrocavitary	60 (42.3)
Lamellar shadow	134 (94.4)
Nodule	111 (78.2)

TB, tuberculosis; COPD, chronic pulmonary obstructive disease; HIV, human immunodeficiency virus; AFB, acid-fast bacilli.

The data are presented as number (%) unless otherwise specified.

**Table 3.** Comparison between male and female patients with pulmonary NTM disease (*N* = 142)

Characteristics	Male (*N* = 88, %)	Female (*N* = 54, %)	*χ*^2^	*P* value
Comorbidities				
TB history	23 (26.1)	9 (16.7)	1.719	0.219
Bronchiectasis	32 (36.4)	15 (27.8)	1.114	0.359
Clinical presentations				
Cough	82 (93.2)	43 (79.6)	5.832	0.030*
Haemoptysis	33 (37.5)	11 (20.4)	4.592	0.040*
Laboratory investigations				
AFB smear (+)	22 (25.0)	12 (22.2)	0.142	0.840
Chest X-ray				
Fibrocavitary	41 (46.6)	19 (35.2)	1.784	0.222
Lamellar shadow	84 (95.5)	50 (92.6)	0.516	0.479
Nodule	67 (76.1)	44 (81.5)	0.560	0.533

TB, tuberculosis; AFB, acid-fast bacilli.

The data are presented as number (%) unless otherwise specified; categorical variables were tested by *χ*^2^ test to assess the difference between male and female patients with pulmonary NTM disease.

### Drug resistance characteristics of the NTM species

Of the 146 patients with NTM disease, 108 were tested for antimicrobial susceptibility (results are shown in Table 4). For rapidly growing non-tuberculous mycobacteria (RGM), linezolid was the most highly active agent against *M. abscessus complex* and *M. fortuitum*, and only one of the *M. abscessus complex* isolates (1.9%) was resistant to linezolid. Clarithromycin and amikacin also showed potent activity against *M. abscessus complex* and *M. fortuitum*, and <12% of the *M. abscessus complex* and *M. fortuitum* were resistant to clarithromycin and amikacin. In addition, tobramycin showed low activity against *M. fortuitum*, and the percent of tobramycin-resistance was observed in 76.5% of the *M. fortuitum* isolates. For slowly growing non-tuberculous mycobacteria (SGM), amikacin and linezolid were completely sensitive to *M. intracellulare* and *M. avium*. *Mycobacterium kansasii* was found to be sensitive to all of the tested antimicrobial agents.

**Table 4.** Number of NTM clinical strains resistant to drugs in *in vitro* experiments (*N* = 108)

Species/complex	Antimicrobial agents						
	CLAR	AMK	MOX	LZD	CFX	TBM	
RGM							
*M. abscessus complex* (*N* = 53, %)	4 (7.5)	2 (3.8)	20 (37.7)	1 (1.9)	7 (13.2)	22 (41.5)	
*M. fortuitum* (*N* = 17, %)	2 (11.8)	1 (5.9)	1 (5.9)	0 (0.0)	3 (17.6)	13 (76.5)	
Species	Antimicrobial agents						
	CLAR	AMK	MOX	LZD			
SGM							
*M. intracellulare* (*N* = 27, %)	4 (14.8)	0 (0.0)	4 (14.8)	0 (0.0)			
*M. avium* (*N* = 8, %)	1 (12.5)	0 (0.0)	2 (25.0)	0 (0.0)			
	CLAR	AMK	MOX	LZD	RIF	MIN	RFB
*M. kansasii* (N = 3, %)	0 (0.0)	0 (0.0)	0 (0.0)	0 (0.0)	0 (0.0)	0 (0.0)	0 (0.0)

RGM, rapidly growing non-tuberculous mycobacteria; SGM, slowly growing non-tuberculous mycobacteria; CLAR, clarithromycin; AMK, amikacin; MOX, moxifloxacin; LZD, linezolid; CFX, cefoxitin; TBM, tobramycin; RIF, rifampicin; MIN, minocycline; RFB, rifabutin.

The data are presented as number (%) unless otherwise specified.

## Discussion

To the best of our knowledge, this is the first systematic, large-scale study conducted in Chongqing to investigate the occurrence of NTM disease. Our data demonstrate that pulmonary NTM disease was the most prevalent one in NTM diseases in Chongqing. Eight NTM species were isolated, and among them, *M. abscessus complex* was the most commonly detected one. Male patients were more prone to cough and haemoptysis than the female ones. Linezolid, amikacin and clarithromycin exhibited favourable activity against most of the species, while tobramycin exhibited only limited activity against *M. fortuitum*.

The distribution of the NTM species tended to vary with the geographical location. In this study, *M. abscessus complex* was discerned to be the most prevalent one, based on an observation that was consistent with those of previous reports from Singapore and a study conducted in the subtropical regions of Japan [15, 16]. However, *M. avium* was the most isolated species in patients with pulmonary NTM disease in Taiwan, Canada, Portland and the USA [9, 17, 18], which was only the fifth-most common causative agent in our region. *Mycobacterium fortuitum* was the third-most frequently isolated NTM species and was detected in 11.7% of the patients in the current study. Although the role of *M. fortuitum* in NTM disease remains controversial [19], previous studies have reported it to be a causative agent of pulmonary disease [20]. Moreover, *M. fortuitum* is the second-most common cause of rapidly growing mycobacterial infections in China [21]. The differences in the NTM species distribution can be partly attributed to variations in the sample sources.

The clinical significance of different NTM species being isolated from the same patient remains unclear, but it is understood to be one of the key features of NTM lung infection [22, 23]. In this study, only 7.5% of the patients with NTM disease harboured ≥2 NTM species, but the proportion was much higher (30.1%) in a previous study conducted in Singapore [15]. Zhang *et al*. claimed that the age of ≥65 years and COPD were significantly associated with multispecies isolation [15]. The multispecies infection may be associated with the changing of the clinical situation, as also suggested by Lee *et al*. [24]. Nonetheless, as data pertaining to clinical treatment were not collected, the correlation between multispecies infection and clinical outcome could not be ascertained. Hence, the clinical implications of multispecies isolation need to be further elucidated. The definitive identification of single or mixed mycobacterial infection must be emphasised to shed more light on this issue.

Certain studies have indicated that pulmonary NTM disease affects women more frequently than men [8, 22]. On the other hand, early reports from the USA and Europe have suggested that the disease is more strongly associated with men [25, 26]. In our study, 62% of the patients were men. Several studies have reported that this condition could be attributed to structural lung diseases [9, 15]. In agreement, 40% of the patients in this study had either COPD or bronchiectasis, with a higher proportion of the subjects experiencing bronchiectasis (33.1%). Pulmonary NTM disease and bronchiectasis are inextricably linked [27]. In fact, it has been hypothesised that the impaired mucus clearance due to bronchiectasis increases the NTM airway colonisation and the risk of infection [28]. However, contrary to some previous studies that reported bronchiectasis to be more common among female [9, 15], we did not find any significant difference between the male and female patients in this regard. Pulmonary TB has been reported to be associated with pulmonary NTM disease as it also involves severe pulmonary structural damage [29]. In this study, about one-fourth of the patients with pulmonary NTM disease demonstrated a history of TB. According to our results, cough and haemoptysis were the common symptoms of pulmonary NTM disease, with a much higher incidence in the male patients. This result is contrary to the study by Zhang *et al*., in which haemoptysis was more likely to affect female patients with pulmonary NTM disease [15]. The differences may be partially related to the difference in the study population.

Macrolides (clarithromycin, azithromycin, roxithromycin and erythromycin) are regarded as effective drugs in the treatment of NTM infections, and clarithromycin is the most active agent in this category [30]. Our results indicated the potential efficacy of clarithromycin in controlling the infection. Here, we have grouped the *M. abscessus complex* (including *M. abscessus*, *M. massiliense* and *M. bolletii*) together as the three species could not be reliably differentiated by the species identification kit used. These three species differed in their susceptibility to macrolides owing to the presence of a functional *erm* gene or its truncation [31, 32]. Thus, further study is warranted to accurately identify the *M. abscessus complex* at the subspecies level. In case of aminoglycosides, amikacin showed significant antimicrobial activity against almost all NTM species. However, the results from another study imply that amikacin resistance in NTM patients is quite common [33]. This discrepancy may be associated with the regional diversities between the subspecies. Furthermore, all our isolates were susceptible to linezolid, except for the *M. abscessus complex* isolates. Therefore, linezolid is a potentially good choice for treatment regimens against the *M. abscessus complex*, *M. fortuitum*, *M. intracellulare*, *M. avium* and *M. kansasii*. Accurate species identification and drug susceptibility testing are imperative to ensure appropriate treatment modality for combating NTM infection.

This study has several limitations. First, it was conducted in a single hospital in Chongqing. Nevertheless, since the Chongqing Public Health Medical Centre is the biggest and the most specialist hospital for TB and other infectious diseases, it has certain representativeness for the incidence of NTM diseases in the entire Chongqing region. Second, the molecular mechanisms of drug resistance were not investigated in this study, necessitating further experiments in the future.

In conclusion, our results indicate that the prevalence of NTM infection in Chongqing is common and that the dominant causative species is *M. abscessus complex.* Hence, clinicians should consider the possibility of NTM infection in case of TB-like symptoms. As susceptibility to antibiotics is highly species-specific, the identification of the causative agent and drug susceptibility testing play a pivotal role in a successful treatment. Further studies are needed to decipher the molecular mechanisms of resistance toward specific antibiotics to develop appropriate treatment strategies in time.

## Data

The data in this study are available from the first author (H.Z.) on reasonable request.
